# End-of-life care preferences among cancer patients in Southern Thailand: a university hospital-based cross-sectional survey

**DOI:** 10.1186/s12904-021-00775-6

**Published:** 2021-06-23

**Authors:** Jarurin Pitanupong, Sahawit Janmanee

**Affiliations:** Department of Psychiatry, Faculty of Medicine, Prince of Songkla University, 90110 Hat Yai, Songkhla Thailand

**Keywords:** Cancer, Care, End of life, Patient, Preference

## Abstract

**Background:**

End-of-life care preferences may be highly individual, heterogenic, and variable according to culture and belief. This study aimed to explore preferences and factors associated with end-of-life care among Thai cancer patients. Its findings could help optimize the quality of life of palliative cancer patients.

**Methods:**

A cross-sectional study surveyed palliative cancer outpatients at Songklanagarind Hospital from August to November 2020. The questionnaires inquired about: (1) personal and demographic information, (2) experiences with end-of-life care for their relatives, and (3) end-of-life care preferences. To determine end-of life preferences, the data were analyzed using descriptive statistics. The data concerning patient demographics and end-of-life care preferences were compared using Fisher’s exact test.

**Results:**

The majority of the 96 palliative cancer outpatients were female (65.6 %), and the overall mean age was 55.8 ± 11.6 years. More than half of them had an experience of observing someone die (68.8 %), and they were predominantly being conscious until the time of death (68.2 %). Most participants preferred receiving the full truth satisfied with the care their relatives had received in passing away at home surrounded by family (47.0 %) and regarding their illness (99.0 %), being free of uncomfortable symptoms (96.9 %), having their loved ones around (93.8 %), being mentally aware at the last hour (93.8 %), and having the sense of being meaningful in life (92.7 %). Their 3 most important end-of-life care wishes were receiving the full truth regarding their illness, disclosing the full truth regarding their illness to family members, and passing away at home.

**Conclusions:**

In order to optimize the quality of life of palliative cancer patients, end-of-life care should ensure they receive the full truth regarding their illness, experience no distressing symptoms, remain mentally aware at the last hour of life, feel meaningful in life, and pass away comfortably with loved ones around.

## Background

According to WHO, cancer is one of the leading causes of death worldwide. In 2018, a study found that around 9.6 million people die from cancer every year, and the number of people dying from cancer is on the rise. Cancer patients suffer from both the disease and the related therapeutic processes, especially during the terminal stage [1]. Therefore, for patients who have a life-threatening disease and a life expectancy of 6 to 12 months, the care process should focus on improving the quality of life during their end-of-life (EoL) stage in order to assist patients to die in peace and comfort, and with dignity [2].

During the past decade, awareness on EoL care has increased, and the concepts related to it have changed over time [3–6]. Across cultures and ethnicities, being free from pain and shortness of breath, not being a burden to one’s family, and feeling that one’s life is completed have been rated as the core components of EoL care [7]. However, EoL care preferences are highly individual and heterogenic and vary widely according to one’s culture and beliefs [4, 6].

In the United States of America, a study found that the most important EoL care preferences among terminally ill patient’s were: being mentally aware until the last hour; having funeral arrangements planned; not being a burden to the family or society; being able to help others [8]; dying in one’s sleep; and experiencing a quick, painless, and peaceful death, without suffering and while praying [8, 9].

In the EU, a study in Sweden identified these EoL care preferences among adult palliative cancer patients: living with the prospect of imminent death, preparing for death, and dying comfortably [4]. Besides, various studies from Asia, South Korea, China, and Japan have reported these 4 most important components regarding EoL care preferences: not being a burden to others, having a good relationship with family members, having a good relationship with medical staff, and being free from physical and psychological discomfort [10–12].

Some associated factors that lead to differences in EoL care preferences are age, religion, occupation, education, economic status, being married, having a family or caregiver, current serious illness, history of hospitalization, being a caregiver for a seriously ill patient, having the experience of a closed one dying, and self-satisfaction [11, 13]. As reported previously, there are some different aspects regarding EoL care preferences between Asian and Western countries. The topics of death and dying could be discussed more freely in the West than in Asia. Moreover, in Asians societies, the family is more involved in decision-making related to EoL care, and Asians choose to die at home more frequently than Western people [1]. Hence, understanding the patients’ EoL care preferences is key to succeeding in improving their quality of life. If healthcare providers do not have a firm grasp on these issues, the caring process that aims to promote a good quality of life for patients may be compromised [6, 14].

In Thailand, palliative care has been developing since the late 1990 s. Initially, it had slow progress during the first decade of development. Recently, it has seen rapid growth and received attention from both government and non-government organizations. However, there remains a long way to move forward to reach a high standard and excellence of care for all persons at the end of life [15]. Regarding these issues, there were workshops on peacefully facing death for more than 10 years and published several books on death and dying issues. Also, a study conducted among Thai elderly patients from the Central and Northeastern regions in 2017 revealed that their 3 most important EoL care preferences were receiving the full truth about their illness, passing away at home and having loved ones around at the time of death, and receiving relief from symptoms such as pain and shortness of breath [13]. However, while the majority of Thailand’s population are Buddhist, many provinces in the Southern Region were predominantly Muslim. Therefore, Southern peoples’ beliefs might differ from those of Thais from the Central and Northeastern regions. To our knowledge, no study on EoL care preferences has been conducted in the Southern Region of Thailand over the past decade. Therefore, this study aimed to evaluate EoL care preferences for themselves and their relatives, their associating factors among palliative cancer patients from the South in order for its findings to provide basic knowledge that may be useful for the employment and/or establishment of a realistic and effective caring process through various psychosocial support frameworks before, during, and after the dying period that would ensure a better quality of life for both patients and their loved ones.

## Methods

 After being approved by the Ethics Committee of the Faculty of Medicine, Prince of Songkla University (REC: 63-099-03-4), this cross-sectional study was conducted at Songklanagarind Hospital, which is an 800-bed university hospital that serves as a tertiary referral center in Southern Thailand. All palliative cancer patients treated at the Radiotherapy Clinic from August to November 2020 were invited to participate in this study. To be included, one had to meet the criteria of being a palliative cancer outpatient by the owner physician and was retrieved from the medical register, aged more than 18 years, being able to understand and use the Thai language well, acknowledging his/her cancer diagnosis, agreeing to participate in the study, and completing all of the questionnaires. Meanwhile, those who were unaware of their cancer diagnosis, lacked the mental capacity to complete all of the questionnaires, and did not wish to participate or decided to withdraw from the study were excluded.

In respect to the sample size calculation, the literature review suggested that the proportion of patients that agreed with each item of EoL care preference had to be at least 50.0 % [1]. To ensure an appropriate proportion estimation, the researchers calculated the sample size with the consideration of a margin of error value of 10 %. A p-value of < 0.05 was determined to indicate statistical significance. Hence, a sample of at least 96 subjects was deemed necessary.

The researchers approached all of the potential palliative cancer outpatients for recruitment and handed them an information sheet, which delineated the rationale for the study and the allotted time to complete the survey. They had at least 10–15 min to consider whether to collaborate in the study or not. If they wished to participate, all of them was asked to sign the informed consent form and was invited to a private location to complete the questionnaires. The researcher observed the participants’ reactions and informed them that they could stop at any time if they felt uneasy, distressed, or were unwilling to participate any further. Moreover, if the participants exhibited a high level of distress or worry, advice or further clinical management was provided to them.

### Measures

 The questionnaires were reviewed by 5 psychiatrists (one of them practiced in the Palliative Care Unit of our hospital), and a content validity was performed; the content validity index (CVI) score was 0.8. A pilot study was conducted on 25 participants, and the Cronbach’s alpha was 0.8. The questionnaire was composed of 3 parts:

1. Personal and demographic information inquiry consisting of questions related to age, gender, marital status, religion, income, education, hometown, number of household members, history of substance use, physical and psychiatric illness, history of hospital admission, and perception concerning their satisfaction with their health and life.
2. Inquiry regarding experiences with EoL care for their relatives, which consisted of 4 questions on experiences related to seeing relatives die and being an EoL caregiver. Each item was rated as ‘yes’ or ‘no’; the attitude toward experiences with the death of a relative was rated as ‘satisfied’, ‘unsatisfied’, or ‘neither satisfied nor unsatisfied’, meanwhile the attitude toward the relatives being remembered after their death was rated as ‘agree’, ‘disagree’, or ‘neither agree nor disagree’ [1].
3. Inquiry on EoL care preferences focusing on the sort of end-of-life care they would like for themselves, which consisted of 2 categories (total 15 items) [1, 13].

3.1) Importance of EoL care comprised the 12 following items: receive the full truth about their illnesses, disclose the truth about their illnesses to family members, have loved ones around when needed, not be a burden to the family, complete unfinished business and be prepared to die, feel that life is meaningful, receive relief from distressing symptoms, receive both physical and psychological treatment, participate in and perform religious rituals, be involved in treatment decisions, be mentally aware at the last hour, and pass away at home.
3.2) EoL care preferences consisted of the 3 following items: withhold of futile life-sustaining treatment, administration of active pain control, and performance of active euthanasia.

The scores of all the 15 questions ranged from 1 to 5 (strongly agree, agree, disagree, strongly disagree, and neither agree nor disagree). The responses of ‘strongly agree’ and ‘agree’ were classified as indicating approval for the intervention [13]. Furthermore, the participants were asked to rate the 3 most important components among the 15 items.

### Statistical methods

Descriptive statistics such as proportion, mean, and standard deviation (SD) were calculated. The data related to patient demographics and end-of-life care preferences were compared using Fisher’s exact test. The statistical significance was defined as a p-value of less than 0.05.

## Results

### Demographic characteristics

 From August to November 2020, 96 palliative cancer outpatients participated in the survey by completing the constructed questionnaires. The majority of them were female (65.6 %), married (80.2 %), and Buddhist (72.9 %). The mean age was 55.8 ± 11.6 years, and the median income (IQR) was 20,000 (10,000–37,500) baht per month. In addition, most participants were satisfied with their health (73.9 %) and reported no history of substance use, physical illness (excluding their cancer diagnosis), or psychiatric illness (89.6 %, 63.5 %, 97.9 %, respectively). The most common physical diseases were hypertension (18.8 %), diabetes mellitus (12.5 %), and dyslipidemia (10.4 %). Moreover, most participants were satisfied with their life (83.3 %) (Table 1).

**Table 1:** Demographic characteristics (*n* = 96)

Demographic characteristics	Number (%)
**Gender**	
Male	33 (34.4)
Female	63 (65.6)
**Marital status**	
Single/divorced	19 (19.8)
Married	77 (80.2)
**Religion**	
Buddhism	70 (72.9)
Islam, Christianity	26 (27.1)
**Education**	
Primary school and below	39 (40.6)
Secondary school	28 (29.2)
Bachelor’s degree and above	29 (30.2)
**Hometown**	
Songkhla Province	25 (26.0)
3 Southern Border Provinces	21 (21.9)
Other	50 (52.1)
**Physical illness (excluding their cancer diagnosis)**	
No	61 (63.5)
Yes	35 (36.5)
**Psychiatric illness**	
No	94 (97.9)
Yes	2 (2.1)
**History of substance use**	
No	86 (89.6)
Yes	10 (10.4)
**Number of household members**	
Alone	5 (5.2)
Less than 3	15 (15.6)
More than 3	76 (79.1)
**History of admission**	
Yes	46 (47.9)
No	50 (52.1)
**Satisfaction with own health**	
Excellent	3 (3.1)
Good	29 (30.2)
Fair	39 (40.6)
Poor	25 (26.0)
**Satisfaction with life**	
Satisfied	80 (83.3)
Unsatisfied	16 (16.7)

### Experiences with and attitude towards the end-of-life care for their relatives

Sixty-six participants (68.8 %) reported having had the experience of seeing a loved one die, and half of them had had the role of an EoL caregiver. The majority of them were satisfied with the end-of-life care their relatives had received in respect to passing away at home with family members around (47.0 %) and being remembered after death (68.2 %). However, concerning the issues of dying in the hospital and resuscitation being administered or withheld, the majority of participants failed to give an opinion either way (66.7 and 69.7 %, respectively) (Table 2).

**Table 2:** Experiences with and attitude towards the end-of-life care for their relatives (*n* = 66)

Type of EoL care	Number (%)
**Passing away at hospital with CPR**	
Good	8 (12.1)
Bad	14 (21.2)
No opinion	44 (66.7)
**Passing away at hospital without CPR**	
Good	14 (21.2)
Bad	6 (9.1)
No opinion	46 (69.7)
**Passing away at home among family**	
Good	31 (47.0)
Bad	15 (22.7)
No opinion	20 (30.3)
**Attitude toward being remembered after death**	
Good	45 (68.2)
Bad	14 (21.2)
No opinion	7 (10.6)

### End-of-life care preferences

In regard to the importance of EoL care, most participants identified five major caring components as the most important ones: receiving the full truth about their illnesses (99.0 %), being relieved from distressing symptoms such as pain and shortness of breath (96.9 %), having loved ones around when needed (93.8 %), being mentally aware until the time of death (93.8 %), and having the sense of being meaningful in life (92.7 %). However, passing away at home was rated as the least important component (61.5 %), and one-third of them found it difficult to give an opinion about this component (31.2 %) (Table 3).

**Table 3:** End-of-life care preferences (*n* = 96)

EoL care preference	Number (%)
**Importance of EoL care aspects**	
Receiving the full truth regarding their illness	
Disagree	1 (1.0)
Agree	95 (99.0)
No opinion	-
Disclosing the full truth regarding their illness to family members	
Disagree	7 (7.3)
Agree	88 (91.7)
No opinion	1 (1.0)
Having loved ones around when needed	
Disagree	3 (3.1)
Agree	90 (93.8)
No opinion	3 (3.1)
Not being a physical or psychological burden to the family	
Disagree	22 (22.9)
Agree	69 (71.9)
No opinion	5 (5.2)
Completing unfinished business; preparing to die	
Disagree	12 (12.5)
Agree	83 (86.5)
No opinion	1 (1.0)
Having the sense of being meaningful in life	
Disagree	3 (3.1)
Agree	89 (92.7)
No opinion	4 (4.2)
Being free from distressing symptoms such as pain and shortness of breath	
Disagree	2 (2.1)
Agree	93 (96.9)
No opinion	1 (1.0)
Receiving both physical and psychological treatment	
Disagree	6 (6.2)
Agree	89 (92.7)
No opinion	1 (1.0)
Performing or participating in religious rituals	
Disagree	16 (16.7)
Agree	73 (76)
No opinion	7 (7.3)
Being involved in treatment decisions	
Disagree	15 (15.6)
Agree	77 (80.2)
No opinion	4 (4.2)
Being mentally aware at the last hour of life	
Disagree	4 (4.2)
Agree	90 (93.8)
No opinion	2 (2.1)
Passing away at home	
Disagree	7 (7.3)
Agree	59 (61.5)
No opinion	30 (31.2)
**EoL care preferences**	
Withhold of futile life-sustaining treatment	
Disagree	19 (19.8)
Agree	74 (77.1)
No opinion	3 (3.1)
Active pain control	
Disagree	8 (8.3)
Agree	87 (90.6)
No opinion	1 (1.0)
Active euthanasia	
Disagree	38 (39.6)
Agree	57 (59.4)
No opinion	1 (1.0)

Regarding EoL care preferences, most participants reported a high level of preference for active pain control (90.6 %) (Table 3). Moreover, the 3 most important reported components regarding EoL care wishes were: receiving the full truth about their illnesses, disclosing the full truth concerning their illnesses to the family, passing away at home with loved ones around when needed, and being mentally aware until the last hour. Moreover, receiving relief from symptoms such as pain and shortness of breath was also highly preferred.

### Association between demographic characteristics, experiences related to EoL care for relatives, and EoL care preferences

We tried to discover the factors that associate with EoL care preferences. However, the majority of responses were in agreement with one another, so no differences between the participants could be calculated. Therefore, based on this dataset, we failed to determine any factors associated with EoL care preferences.

## Discussion

 This is the first study from Southern Thailand that surveyed the EoL care preferences of palliative cancer patients. The five most important components of EoL care discovered were: receiving the full truth about their illnesses, being relieved from distressing symptoms such as pain and shortness of breath, having loved ones around when needed, being mentally aware until the time of death, and feeling meaningful in life. Furthermore, the participants placed a high level of preference on receiving the full truth about their illnesses, disclosing the full truth about their illnesses to the family, passing away at home with loved ones around when needed, and being mentally aware until the time of death. These findings support those of prior reports from the United States of America [8, 9] and the EU [4] as well as a study conducted among Thai elderly patients from the Central and Northeastern regions of Thailand in 2017 [13]. A reason for this might be the fact that most participants in this study were older adults with a mean age of 55.8 years. Also, knowing the full truth about one’s illnesses, being mentally aware until the time of death, preparing for death, and dying peacefully while feeling comfortable and without pain or suffering might be universal human need or basic core EoL care preferences among palliative cancer patients [4, 8, 9]. Therefore, there were no diversity and cultural difference.

Almost all participants identified ‘receiving the full truth about their illnesses’ as their number one need in regard to EoL care. Moreover, being mentally aware at the last hour of life was one of the five top EoL care preferences. This indicates that autonomy was of a critical importance to our palliative cancer patient population. However, since ‘disclosing the full truth regarding their illnesses to family members’ was a significant preference, it could be said that they were also likely to involve their family in the decision-making process related to their EoL care. This finding is consistent with those of a prior study on Northern Thai patients with a terminal illness; they desired that both physicians and relatives be involved in deciding the EoL care they received [13]. Furthermore, this finding supports the report of a former study that Asian patients are more likely to have their family involved in medical decision-making rather than exercising full autonomy or decide only by themselves like most American and European patients do [1, 16, 17]. Concerning this matter and in light of this finding, Thai physicians should ask their patients whether they wish to receive information regarding their health conditions and treatment and to what extent, as well as whether they would like to be involved in making decisions related to their care or prefer that their families handle such matters [18]. However, regarding resuscitation being administered or withheld for their relatives, this study showed the majority of participants failed to give an opinion either way. Then, all Thai palliative cancer patients should be promoted to exercise full autonomy and have a chance to adjudge the end-of-life procedures by themselves.

In relation to being relieved from distressing symptoms, the participants identified that ‘being free from symptoms such as pain and shortness of breath’, which cause considerable suffering, was the second important component of EoL care. Once more, this finding is consistent with those of a prior study. They found that even before the EoL stage, nearly half of their cancer patients reported moderate-to-severe pain; up to 30.0 % identified their pain level as severe, and an estimated 25.0 % died in pain [19]. Therefore, all physicians should make the exploration and relief of their patients’ distress and suffering a priority of medical care. Moreover, knowledge regarding pain, basic end-of-life pain management, and symptom control should be offered to palliative cancer patients and their families [20–22].

Regarding passing away, a good death is one that is free from avoidable suffering, for the patient, their relatives, and caregivers alike; in general accord with the patient’s and family’s wishes; and reasonably consistent with clinical, cultural, and ethical standards [10, 23]. Prior studies have identified some factors that influence the passing away process; they are: the location of patient domicile, previous occupation, educational level, family income, family size, and dissatisfaction with life [13, 24]. Moreover, it is known that most elderly Thai patients prefer dying at home [13]. However, this study revealed that only two-thirds of the participants agreed with passing away at home with loved ones around when needed and being mentally aware at the time of death, whereas one-third of them had no opinion on this matter. It seems that dying with meaning and being remembered after death may be more significant than the place of death for some patients [25]. For cancer patients and their families, palliative home care might be difficult to manage due to factors such as the low level of home care support received by the public health system and weakened family networks [26, 27]. Hence, the choice to die in a hospital might be more preferable for the families of palliative cancer patients [24]. However, the preference for dying at home is greater when the patient lives with a caregiver, and the family physician makes home visits [28]. Therefore, both physicians and caregivers should pay attention to the patients’ desires regarding the care they wish to receive as well as respect their characterization of a meaningful death. Moreover, patient care during the last hour in hospitals should be modified in order to make it as similar to passing away at home as possible.

 Towards achieving a good death, harmony regarding the concept of dying well and one’s attitude toward death and dying is important. Furthermore, a healthy interconnectedness among the family, caregivers, and healthcare providers should be the goal. These refer to the physical (symptom control), social (loved ones’ presence), emotional (sharing emotions), and spiritual (inner peace) dimensions of the process of dying. Moreover, the patient’s awareness until the last hour, acceptance of death, and ability to say goodbye to family and friends meaningfully as well as respecting the patient’s wishes should be ensured [24]. For dying individuals, it will be meaningful to experience love and be understood from family. When healthcare professionals provide a compassionate and peaceful environment that facilitates acceptance and hope, the spiritual life of patients is enhanced [23, 29].

Finally, given the importance of family caregivers and healthcare providers in providing care and their impact on the patients’ dying process, it is necessary to reflect upon how their attitudes and prior experiences influence the care for the dying [24, 29]. Hence, a good relationship with family members and healthcare providers improves the patient’s level of self-satisfaction, makes the avoidance of death less likely and the acceptance of death more possible, and enhances their feeling of interconnectedness [13, 29]. These would go a long way in optimizing the terminally ill patients’ quality of life during their EoL period.

To our knowledge, this is the only study on this topic conducted in Southern Thailand during the past decade. However, the study was quantitative, and its sample size was small and restricted to only palliative cancer outpatients in lower Southern Thailand. Hence, its findings may not represent the situation of cancer patients in the whole country fairly. Even though no novel knowledge was obtained from this study, its findings confirmed that there were no differences in end-of-life care preferences between patients from the Southern, Northern and Central regions of Thailand. Henceforward, future studies should include a larger number of palliative cancer outpatients with gender and age-group differences from other hospitals in Thailand; in other words, multi-center research that aims to explore this research topic should be conducted. Moreover, such studies should employ a more qualitative approach or in-depth methodology that is adept at exploring specific disorders.

## Conclusions

The care for palliative cancer patients that is focused on optimizing their quality of life during the end-of-life period should ensure that the patients receive the full truth about their illnesses, be free of distressing symptoms, remain mentally aware until the last hour of life, feel important in life, and pass away comfortably with loved ones around. Further study encompassing other areas or settings in Thailand and employing a more qualitative approach is recommended.

## Data Availability

The datasets used and/or analyzed during the current study can be made available by the corresponding author upon reasonable request.

## Abbreviation

EoL: End-of-life
